# Case report: repair of eventration of the diaphragm in an octogenarian

**DOI:** 10.1093/jscr/rjad581

**Published:** 2023-10-25

**Authors:** Ninon Forter-Chee-A-Tow, Alan Smith

**Affiliations:** Department of Cardiovascular Services, Queen Elizabeth Hospital, Bridgetown 11123, Barbados; Department of Cardiovascular Services, Queen Elizabeth Hospital, Bridgetown 11123, Barbados

**Keywords:** diaphragmatic eventration, diaphragmatic plication, thoracoscopic surgery

## Abstract

Eventration of the diaphragm is a cephalad displacement of the diaphragm because of congenital or acquired causes. The diaphragm maintains its anatomical continuity and normal attachments. It may be partial or complete and unilateral or bilateral. Most adult presentations are asymptomatic, but patients may present with respiratory, gastrointestinal, or cardiac symptoms. Surgical repair is indicated in the symptomatic patient with the most common being diaphragmatic plication. We present surgical repair of a symptomatic left diaphragmatic eventration in an octogenarian.

## Introduction

Diaphragmatic eventration most commonly manifests in pediatric populations but is reported in adulthood as an incidental finding and because of the presence of symptoms. Diaphragmatic eventration can be described as permanent elevation of the involved diaphragm with preservation of continuity and normal attachments. Eventration can be considered paralytic (acquired) or non-paralytic (congenital/true) with true eventration rarely presenting in adulthood. It occurs unilaterally or bilaterally, involves all or part of the diaphragm, and more commonly involves the left hemidiaphragm with higher incidence reported in males.

Surgical repair is reserved for persons with symptomatic presentation and performed via multiple techniques and approaches including thoracic and abdominal repairs. Repair has shown improvement in symptomatology and postoperative pulmonary function. In this report, we present a combined thoracic technique for repair of diaphragmatic eventration in an octogenarian.

## Case report

**Figure 1:**
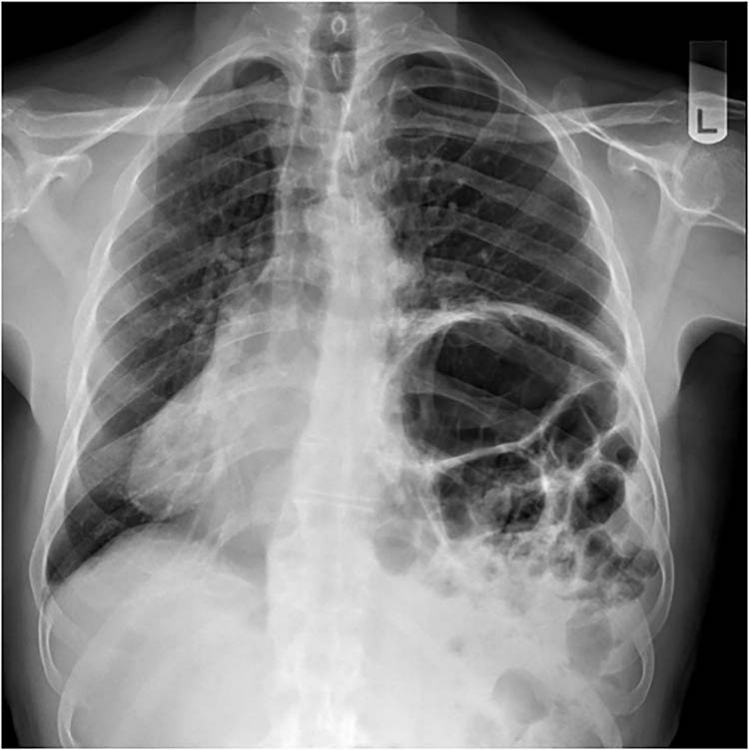
Chest X-ray performed preoperatively.

A 79-year-old male was referred by his general practitioner for consideration for repair of diaphragm eventration. The elevated left hemidiaphragm was noted on chest X-ray approximately a decade before. Referral was prompted by an episode of postprandial shortness of breath and a long-standing inability to belch. Medical history is significant for hypertension and tachyarrhythmia controlled on Flecainide. Clinical examination was remarkable for the presence of bowel sounds in the left chest at the level of the nipple, and apex beat on the right. Chest X-ray showed a markedly elevated left hemidiaphragm with dextrocardia (Fig. 1). Pulmonary function tests (PFT) showed small airway defect with normal lung volumes and diffusion capacity. Chest computed tomography showed no pathology other than the eventration.

**Figure 2:**
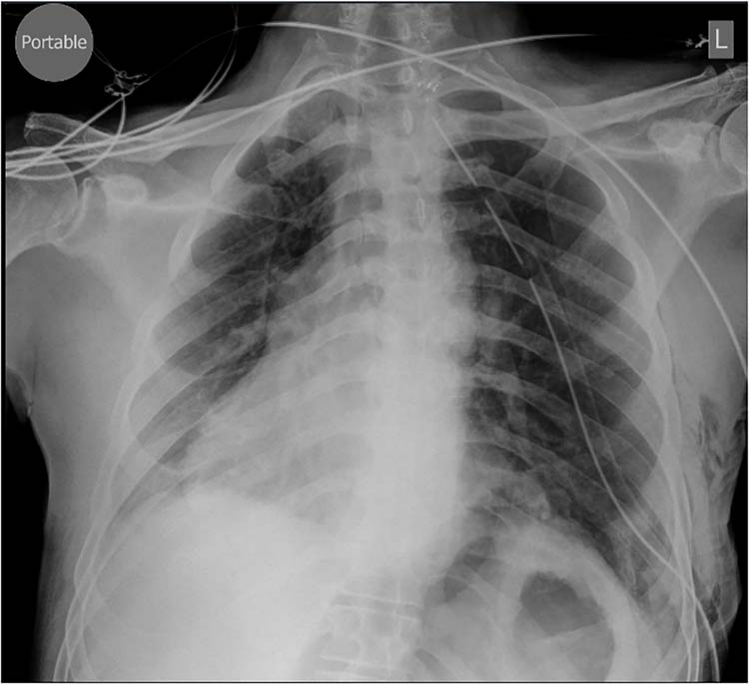
Chest X-ray performed Day 1 postoperatively.

**Figure 3:**
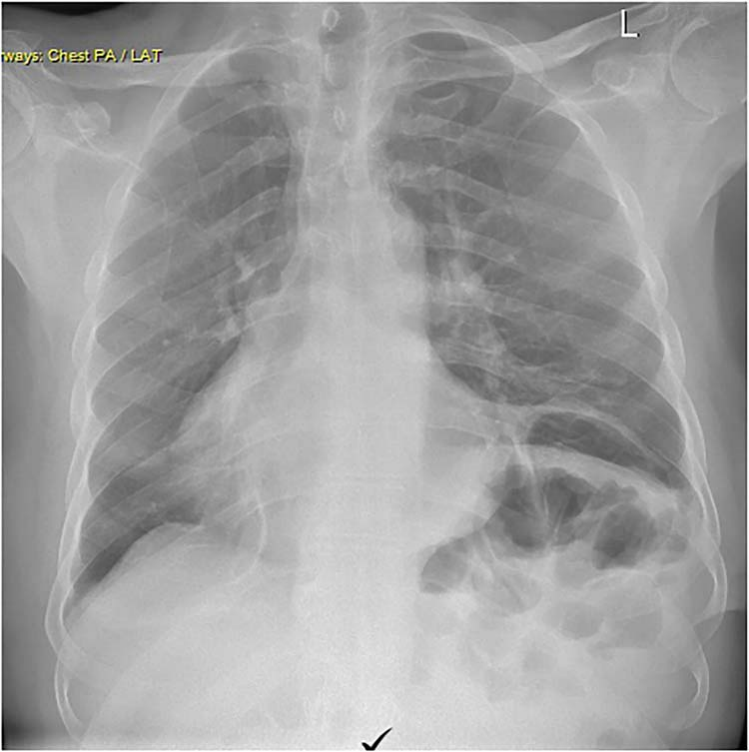
Chest X-ray performed 2 years post-procedure.

Repair was performed via Video-Assisted Thoracoscopic Surgery with an 8 cm access incision and a 5 mm camera port. The central lax portion of the diaphragm was staple resected using an Ethicon stapler followed by plication of remaining redundant diaphragmatic tissue using 2/0 polypropylene running suture. Postoperative chest X-ray showed acceptable descent of the left hemidiaphragm (Fig. 2). Hospital length of stay was 7 days. Postoperative course was complicated by subcutaneous hemorrhage, and development of a left pleural effusion post discharge drained 2 weeks postoperatively by thoracentesis. He also developed severe hiccoughs, settling on a course of esomeprazole. Histology of the 13 × 5 cm resected portion of diaphragm was consistent with eventration. The patient endorsed complete resolution of symptoms at 1 year post-repair and was seen at 2 years post-repair with no issues (Fig. 3).

## Discussion

Diaphragmatic eventration is characterized by permanent cephalic displacement of intact diaphragm with retained continuity and attachments to the costal margins. In its congenital form, there is failure of muscular development in part or all the affected hemidiaphragm because of abnormal migration of cervical myotomes to the septum transversum and pleuroperitoneal membrane. This results in structural deficiency of diaphragmatic muscle with numerous fibrous and fibroelastic changes on microscopic examination.

Prior to our index case, the oldest patient reported in the literature to have undergone surgical repair was a 75-year-old by Watanabe *et al*. [1]. Gastrointestinal, respiratory, and cardiac symptomatology are commonly seen and may not present until adulthood because of weight gain or changes in lung and chest-wall compliance [2]. Dyspnea, orthopnea, epigastric discomfort, palpitations, and symptoms of intermittent intestinal obstruction are among those frequently encountered, often associated with postural changes. Clinical examination may reveal dullness on chest wall percussion, shifting of the apical pulsation, diminished breath sounds, and peristaltic sounds on chest auscultation.

Management of these patients is dictated by presence of symptoms and etiology. While asymptomatic patients often go untreated, there has been renewed discussion on benefits of surgical repair because of longstanding effects on PFT, chronic alveolar hypoventilation, and hypercapnia. Adults with acquired eventration undergo an observation period depending on severity of presenting symptoms prior to definitive repair because of possible improvement in phrenic nerve function. Treatment options include surgical repair by plication with or without a diaphragmatic patch or resection of the redundant segment with overlapping layered tissue repair. Nonsurgical management involves physiotherapy and pulmonary rehabilitation. Higgs *et al*. [3] reported statistically significant data showing improvements in postoperative symptomatology and PFT.

Surgical approaches can be thoracic or abdominal using open or minimally invasive techniques. There is little statistically significant data regarding superiority of thoracic versus abdominal approaches, particularly in adult patients. Pediatric data have shown no statistically significant difference in morbidity and mortality, recurrence, and length of hospital stay between both approaches. Choice of operative technique should be largely based on patient-specific factors. Proposed advantages of laparoscopy include larger working space, reduced risk of visceral injury, and eliminating the need for single-lung ventilation [4]. Relative contraindications to laparoscopy include persons with previous extensive abdominal surgery, body mass index (BMI) > 35, and comorbidities that may worsen with pneumoperitoneum. Reported thoracoscopic techniques for enlarging operative field, such as small incisions for tight trochar fit and CO_2_ insufflation for caudal displacement of intra-abdominal organs, may offer alternatives to proposed laparoscopic advantages [5]. In this case, a thoracic approach was utilized with stapled resection and plication using a running monofilament, nonabsorbable suture.

Proposed complications include pneumonia, pleural effusion such as described in the index case, conversion to open surgical intervention, abdominal visceral injury, pulmonary oedema, chronic pain, and rarely abdominal compartment syndrome [6].

Surgical repair of patients presenting with symptomatic diaphragmatic eventration has shown improvement in symptomatology and pulmonary function [7]. Despite more commonly presenting in the pediatric population, patients may present in adulthood as late as the aforementioned patient [8]. Repair techniques should be tailored to patient factors and institutional practices. This case report highlighted the oldest patient reported in the literature having undergone successful repair.
